# Randomized Clinical Trial to Evaluate the Effect of Probiotic Intake on Androgenic Alopecia

**DOI:** 10.3390/nu16172900

**Published:** 2024-08-30

**Authors:** Alejandro García-Navarro, María Isabel Vasallo-Morillas, Roge Navarro-Belmonte, Cristina Vilanova, Daniel Torrent, Alina Kilasoniya, Isabel Moles-Ugeda, Estefanía Gallego-Herrera, Ana Ramírez-Boscá

**Affiliations:** 1 Centro Dermatológico Estético, 03014 Alicante, Spain; 2 San Antonio Technologies S.L., 30107 Murcia, Spain; 3 Faculty of Medicine, Faculty of Pharmacy and Nutrition, UCAM, Universidad Católica de Murcia, 30107 Murcia, Spain; 4 Darwing Bioprospecting Excellence S.L., 46980 Valencia, Spain; 5 Department of Dermatology, Hospital Universitario del Vinalopó, 03293 Elche, Spain

**Keywords:** androgenetic alopecia, probiotic, gut microbiota, trichoscopy

## Abstract

This study aimed to assess the impact of a combination of probiotic strains of *Lactiplantibacillus* on the treatment of androgenic alopecia (AGA). To this end, 136 individuals with AGA (62 men and 74 women) aged 18–65 years were enrolled in a double-blind, parallel-group clinical trial. A total of 115 individuals (57 in the probiotic group and 58 in the placebo group) completed this study within a 16-week intervention period. Capillary density, thickness, and length of hair were analyzed before and after the intervention using FotoFinder Trichoscale Pro. In addition, the gut microbiota was assessed by paired-end sequencing on the Illumina MiSeq platform (2 × 300 bp). At the conclusion of the treatment period, a notable decline (*p* < 0.05) in the number of telogen hairs was evident in the probiotic group while hair thickness decreased in the placebo group (*p* < 0.05). However, the remaining variables did not exhibit any statistically significant changes. In the probiotic-treated group, individuals aged less than 37.5 years exhibited a reduction in the number and density of telogen hair (*p* = 0.0693 and *p* = 0.0669, respectively) and an increase in hair length (*p* = 0.0871). Furthermore, a notable decline in the number and density of vellus hair (*p* < 0.05) was observed, and this was accompanied by no change in the hair thickness. The probiotic-treated group exhibited a significantly higher abundance of *Lactobacillus* (*p*-adjusted < 0.05, DEseq2 test) and demonstrated a notable reduction in the number and density of telogen hair, and this was accompanied by an increase in the percentage of anagen hair. The probiotic mixture was well tolerated by the participants, with a treatment adherence rate of 90%. In light of this study’s limitations, it can be concluded that a mixture of three strains of *Lactiplantibacillus* promotes the presence of terminal follicles, preventing their gradual miniaturization, which is a characteristic of AGA.

## 1. Introduction

Alopecia refers to hair loss, which can be temporary or permanent, localized, or generalized, and it can be of any type or origin [1]. Hair growth is not continuous as each follicle undergoes a cycle of growth and rests in a non-synchronous manner with its neighboring follicles. At the end of each resting phase, the hair shaft falls out, and a new growth phase begins with the formation of new hair. The hair follicle cycle comprises four phases: anagen (growth phase), catagen (transition phase), telogen (shedding phase), and exogen (follicular atrophy) [2].

Androgenetic alopecia (AGA) is the most common type of alopecia, affecting approximately 80% of men and 50% of women [1]. Androgenetic alopecia is primarily caused by genetic and hormonal factors. Hair loss is caused by the action of male hormones or androgens on certain areas of the scalp that are genetically predisposed to the condition. This leads to a progressive decrease in the activity of the hair follicle, resulting in miniaturization and eventual total atrophy of the hair bulb [1,3]. The enzyme 5-alpha reductase converts testosterone to dihydrotestosterone, which negatively affects hair-follicle growth. Thus, inhibition of this enzyme may be a target for AGA therapy [3]. Although there are several options for treating AGA, including pharmacological-, surgical-, or light-based treatments, as well as nutraceuticals to slow or reverse its progression, selecting appropriate therapies for this chronic condition can be challenging [4].

Pharmacological treatments for androgenetic alopecia (AGA) include oral finasteride for men, topical minoxidil for both men and women, and cyproterone acetate for female AGA in androgenization situations [3]. Other drugs and formulations used to treat AGA were considered off label. Low-level light therapy has also been approved for treating AGA [5].

Minoxidil promotes hair regeneration by increasing both hair diameter and density and exerts its greatest effect on the vertex and frontal regions of the scalp by prolonging the anagen phase [6,7].

Finasteride inhibits the enzyme 5-alpha-reductase type II and prevents the conversion of testosterone to DHT [8]. It has been shown to have long-term effectiveness, resulting in significant hair growth and permanent stabilization of hair loss [9].

Androgenetic alopecia can be triggered by various factors, including nutritional deficiencies (such as biotin, zinc, iron, and essential fatty acids) and endocrine disorders (such as thyroid and menopause), stress, as well as reactions to certain medications (such as fluoxetine, amitriptyline, and sertraline), oral contraceptives, anticoagulants, chemotherapy, allopurinol, and immunosuppressants. Recently, researchers have discovered a new factor in the development of alopecia: the intestinal microbiota [10,11]. The metabolic function of the microbiota plays a key role in maintaining homeostasis by synthesizing micronutrients essential for the host, such as vitamin K, vitamin B12, biotin, niacin, and folic acid [12].

Biotin, also known as vitamin B7, is a water-soluble vitamin that is largely dependent on bacterial production. Biotin is an essential nutrient for skin health. Its deficiency is associated with severe dermatological conditions including hair loss [13]. Alterations in the microbiota can trigger the development of alopecia, especially when there is proliferation of intestinal bacteria capable of degrading biotin in the intestinal tract. Therefore, the use of probiotics that improve host intestinal health can help maintain proper intestinal balance and health.

Supplementation with probiotics has been shown to have beneficial effects on patients with alopecia due to improved blood flow to the scalp. The mechanism of action of these substances is not fully understood, but they seem to stimulate the anagen phase, increasing the conversion of vellus hair into terminal hair [13].

A study was conducted on various probiotic species of the lactic acid bacterium *Lactobacillus*. This study found that these species helped maintain intestinal health, reduced cholesterol levels, and exhibited antioxidant and anti-inflammatory activities. Improvement in the blood lipid profile may lead to better peripheral and scalp blood flow. Furthermore, compared with other degrading strains of the same species, the studied species exhibited a deficiency in biotin assimilation, which increased the availability of this nutrient for the host [14,15,16]. Another clinical study evaluated the efficacy of applying a lotion made with a heat-inactivated strain of *Lactiplantibacillus plantarum* to treat hair thinning in men and women for 24 weeks, resulting in an increase in hair density in the telogen phase [17].

Therefore, in relation to AGA, there are currently no known controlled clinical studies that have evaluated the efficacy of food supplements containing probiotics for its prevention or treatment.

This study aimed to assess the impact of a dietary supplement containing the probiotic strains *Lactiplantibacillus plantarum* DCn_07 (CECT 30102), *L. plantarum* DCn_06 (CECT 30103), and *L. pentosus* DCn1_05 (CECT 30104) on patients with untreated androgenetic alopecia.

## 2. Materials and Methods

### 2.1. Study Design

This study involved a randomized, placebo-controlled, and triple-blind clinical trial with the following two parallel study arms based on the product consumed: probiotic or placebo.

The duration of the intervention was 16 weeks considering several factors. First, although the complete hair cycle is longer than 16 weeks, previous research has shown that significant changes in hair loss and hair density can be detectable in periods of 3 to 6 months since the telogen phase lasts 3–4 months. Studies on other food supplements have reported significant preliminary results with interventions of similar duration. In addition, the effects of probiotics on the intestinal microbiota can be manifested in a relatively short period, so it is of great interest to assess the relationship between changes in the microbiota and hair health. However, from a practical perspective, a 16-week study duration improves feasibility and participant adherence, ensuring greater retention and high-quality data collection [18].

The trial was conducted at the Dermatological Aesthetic Center of Alicante (Spain) between November 2020 and August 2021. The study protocol was approved by the Research Ethics Committee of the General University Hospital of Alicante (internal code: 190726). All participants provided written informed consent before the start of this study. This study was conducted in accordance with the guidelines established in the Declaration of Helsinki.

### 2.2. Study Population

The study participants were selected from among patients at the Alopecia Unit of the Dermatological Aesthetic Center of Alicante, Spain. All patients were consecutively included to minimize selection bias. As patients came to the alopecia unit for medical consultation, they were informed about the study. Patients who met all the inclusion criteria, met none of the exclusion criteria, and signed the informed consent form were included in this study. The inclusion criteria were as follows: (1) an age between 18 and 65 years old; (2) men with Male Androgenetic Alopecia grade I-II-III on the Norwood scale; (3) and women with Female Androgenetic Alopecia grade I-II on the Ludwig scale.

The exclusion criteria were as follows: (1) Participants with autoimmune diseases; (2) pregnant women or women of childbearing age who did not use an effective contraceptive method; (3) lactating women; and (4) participants undergoing hypertension treatment. A total of 136 participants were included in this study.

Candidates were required to commit to not modifying their dietary habits, physical activity habits, or smoking habits before being included in this study.

### 2.3. Randomization, Blinding, Intervention, and Compliance

Subjects included in this study were randomly assigned to one of the treatment groups (probiotic or placebo) in a 1:1 ratio using a randomization list generated by Epidat 3.1. A person who was not involved in this study’s development was responsible for concealing the treatment assignment. Blinding was possible because the probiotic and placebo products were packaged in identical opaque bottles with identical labels containing the required clinical trial data, as per the Good Manufacturing Practice standards, and were differentiated only by a randomized code.

The probiotic treatment comprised a mix of strains previously isolated and characterized by Darwin Bioprospecting Excellence in collaboration with CNCE Innovación. The mix consisted of L. plantarum DCn_07 (CECT 30102), L. plantarum DCn_06 (CECT 30103), and L. pentosus DCn1_05 (CECT 30104) at a total CFU count of 5.0 × 108, whereas the placebo comprised maltodextrin and microcrystalline cellulose. The placebo was identical in terms of organoleptic characteristics, with opaque white gelatin capsules. The investigational products were provided by the study sponsor (CNCE Innovación, S.L., Barcelona, Spain).

The participants were instructed to take one capsule daily before breakfast for 16 weeks with a glass of water. To ensure treatment adherence, patients were provided with a notebook to record the date and time of ingestion.

When the study ended, the container was returned to the investigator for independent evaluation of the treatment adherence.

### 2.4. Outcomes Measurements

During this study, two visits were scheduled: one visit at baseline before the intervention (baseline visit) and another after 16 weeks of intervention. All assessments were performed during visits. The primary efficacy measure was the mean difference in the number of hairs per square centimeter between the baseline and after 16 weeks of intervention. The secondary variables measured included the number of relevant hairs per square centimeter, hair length, hair thickness, comparative manual trichograms, scalp sebum measurement, comparative scalp photographs, and metagenomic analysis of the intestinal microbiota. The safety of the investigated products was also evaluated by recording the adverse events that occurred during the study.

#### 2.4.1. Measurement of the Increase in the Number of Hairs per Square Centimeter

FotoFinder Trichoscale Pro was used. This system generates a report that displays the following general parameters: quantification of the number of hairs and hair density (number of hairs/cm^2^); hair-length parameters (percentage of anagen hairs (%); percentage of telogen hairs (%); number of anagen hairs; number of telogen hairs; number of anagen hairs/cm^2^; number of telogen hairs/cm^2^; average length (mm)); and hair-thickness parameters (percentage of terminal hairs (%), percentage of vellus hairs (%), number of terminal hairs, number of vellus hairs, number of terminal hairs/cm^2^, number of vellus hairs/cm^2^, average thickness (mm), and cumulative thickness (mm/cm^2^)).

#### 2.4.2. Demographic Data and Anthropometry

The participants’ age, sex, weight (kg), and height (cm) were documented in their electronic medical records and recorded in a Data Collection Notebook (DCN).

#### 2.4.3. Comparative Manual Trichograms

This study analyzed the proportion of hair in different phases, including anagen (growth), catagen (transition), and telogen (resting or shedding), as well as dystrophic hair (broken hair), before (V1) and after treatment (V2). For this purpose, a manual trichogram was performed by pulling out a sample of hair from each patient (the root was then analyzed under a microscope to identify the different phases). The measurements were conducted by the same investigator to avoid bias.

#### 2.4.4. Measurement of Scalp Sebum

To determine the level of sebum, we analyzed sebum points using photometry with the Se-bumeter^®^ (Skin Diagnostic SD27, Köln, Germany). The result was calculated using a microprocessor and displayed on the screen as micrograms of sebum/cm^2^ on the skin.

#### 2.4.5. Comparative Scalp Photographs

Scalp photographs were taken in a standardized manner at the beginning and end of treatment from the same selected area using a camera (FotoFinder Dermoscope Vexia MC1000S C, Bad Birnbach, Germany). The results were classified using a five-point scale (2: moderate improvement, 1: mild improvement, 0: no change, −1: mild worsening, and −2: moderate worsening) [19] by two investigators (A.G.N and R.N.B.) who were blinded to the assigned treatment.

#### 2.4.6. Gut Microbial Analysis

To investigate the bacterial communities present in the samples, we amplified the hypervariable region V3-V4 of the 16S ribosomal RNA gene from metagenomic DNA using the universal primers 8F (5′-AGAGTTTGATCCTGGCTCAG′-3′) and 1492R (5′-CGGTTACCTTGTTACGACTT-3′). The PCR cycling conditions included initial denaturation at 95 °C for 3 min, followed by 25 cycles of amplification (30 s at 95 °C, 30 s at 55 °C, and 30 s at 72 °C). Amplification was performed using the KAPA HiFi HotStart ReadyMix PCR kit (KK2602) (Roche Diagnostics (Basel, Switzerland)), and a final extension step was conducted at 72 °C for 5 min, as described by Satari et al. (2020) [20]. Next, Illumina sequencing barcoded adaptors from the Nextera XT index kit v2 (FC-131-2001) (llumina, Inc. (San Diego, CA, USA)) were combined with 16S rRNA amplicons. The resulting libraries were then normalized and merged. The indexed amplicon-containing pooled samples were loaded onto a MiSeq reagent cartridge v3 (MS-102-3003) and supplemented with 10% PhiX control to enhance sequencing quality. Finally, the Foundation for the Promotion of Health and Biomedical Research of the Valencian Community (Fisabio) in Valencia, Spain, performed paired-end sequencing using an Illumina MiSeq platform (2 × 300 bp).

Illumina sequencing data were processed using Qiime2 (v. 2022.11.0) [21]. Sequence-quality assessments were performed using Qiime2 plugin demux (v. 2023.5.0). Trimming, joining, chimera removal, and amplicon sequence variant (ASV) detection (>99.9% similarity) were performed using the Qiime2-integrated DADA2 pipeline (v. 2023.5.0). The taxonomic assignment of each sequence variant was determined using the classify-Sklearn module of the feature classifier plugin (v. 2023.5.0) with SILVA (v. 138) as the reference database. Data were analyzed using the phyloseq R package (v. 1.30.0) [22] and visualized using ggplot2 (v. 3.4.0) and ampvis2 (v. 2.7.2) [23]. Beta diversity analysis was conducted using principal component analysis (PCoA) based on Bray–Curtis dissimilarities to evaluate the similarity of the microbial communities.

#### 2.4.7. Safety of the Investigational Products

The safety of the investigational products (PIs) was assessed by recording the adverse events (AEs) throughout the study. These records contain information about the nature of the adverse event, including the diagnosis (if known), signs or symptoms, intensity, start date, end date, and the relationship with the administration of the PI. The actions that were taken to reverse them, including those involving modification of the study treatment, were also documented. Furthermore, we assessed whether they were serious adverse events. This study collected the signs and symptoms of AEs related or unrelated to the investigational products when reported spontaneously by the participant in the patient’s diary by means of a telephone call to the investigator or through biweekly online communication between the investigator and the participant to detect the occurrence of an adverse event, or when the investigator inquired during scheduled visits.

### 2.5. Data-Quality Assurance

Data quality was ensured through study monitoring at the research center. The Contract Research Organization (CRO), San Antonio Technologies S.L. (Murcia, Spain), verified the correct inclusion of participants in this study, as well as the accurate recording of data in the DCN for each participant based on source documents.

### 2.6. Sample Size

The calculation of the sample size (by Epidat 3.1 software) was based on the primary variable, which was the increase in the number of hairs per square centimeter during the 16-week follow-up period. To achieve a precision of 12 hairs/cm^2^, an alpha risk of 5%, and a power of 80%, 55 subjects were required in each group considering a standard deviation of 25.4 hairs/cm^2^ [24]. To account for a 15% dropout rate, 65 participants were required in each group.

### 2.7. Statistical Analysis

The statistical package was “The language and environment for statistical computing, R version 4.3.0”.

At the baseline visit, descriptive analysis of the sample characteristics was performed. This analysis described each variable in each of the groups. Categorical variables were described using proportions, whereas continuous variables were described using means and standard deviations.

Analysis of results: A linear regression model was used to analyze each target variable separately. The dependent variable was the difference between a baseline visit and a follow-up visit of the target variable, whereas the explanatory variables were the treatment group and the target variable at the baseline visit centered on its mean. This methodology allows for the control of the regression-to-the-mean effect. The expected mean change in each group between the two visits was thus calculated, with the baseline value being adjusted for. The treatment effect was calculated as the difference between the two groups’ estimated mean change. Prior to this analysis, the normality of the data was assessed using the Shapiro–Wilk test, and the homoscedasticity was evaluated using Snedecor’s F test or Fligner’s test, as appropriate. When the data were not normal or homoscedastic, the Mann–Whitney U test or robust tests for trimmed means were used, respectively.

For microbiome analyses, we used the Wilcoxon rank-sum test to test for significant differences at the alpha diversity level. PERMANOVA tests were performed using the adonis2 function in the vegan R package (v. 2.6.4) [25] to determine the statistically significant differences in microbiome composition among the analyzed groups. A Wald test was used to perform differential abundance analyses between the taxa using the DESeq2 package in R (v. 1.26.0) [26], with the Benjamini–Hochberg technique used as a method for adjusting the *p*-values.

## 3. Results

### 3.1. Capillary Analysis

**Figure 1:**
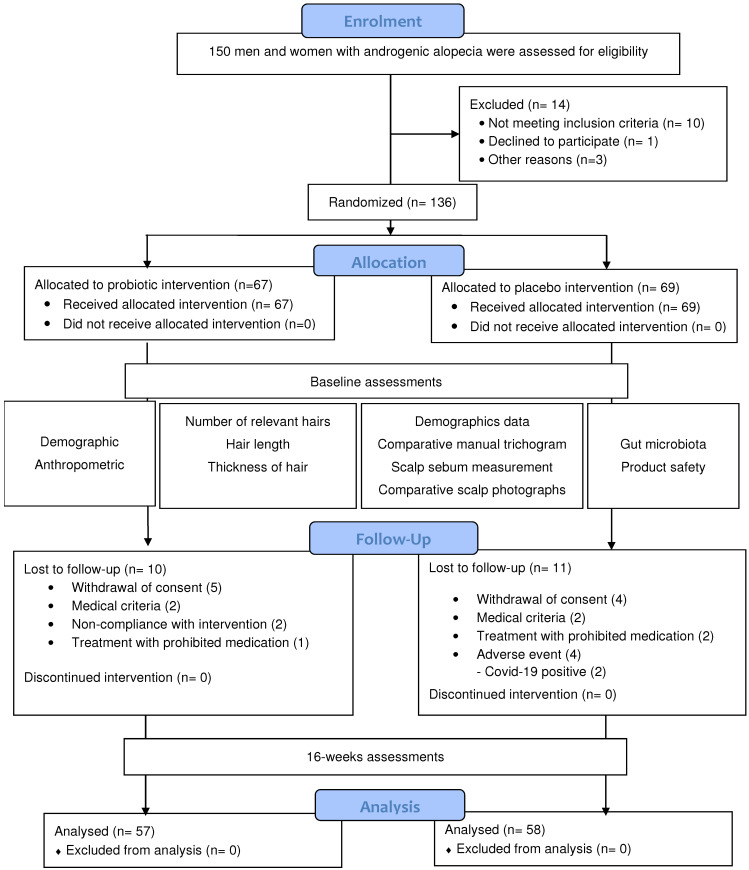
CONSORT diagram of the randomized controlled trial.

Of the 150 patients evaluated, 136 were randomized, of whom 115 completed the study (Figure 1). The placebo group comprised 34 men and 35 women, whereas the probiotic group comprised 28 men and 39 women. The mean age was 38.2 ± 11.3 years in the probiotic group and 39.4 ± 10.8 years in the placebo group.

The number and density of total hair did not change significantly at the end of treatment for any of the groups under study, nor was there any difference between them. There was also no significant change in the number and density of anagen hair. However, at the end of the probiotic treatment, the number of telogen hairs significantly decreased (*p* < 0.0314) (Table 1).

As shown in Table 2, the mean hair length did not change significantly in the study participants, but the mean thickness was reduced in the placebo-treated group (*p* = 0.0301), whereas probiotic-treated individuals showed no change in hair thickness (*p* = 0.6842). The difference between the two groups (placebo and probiotic) for this parameter did not meet the threshold of significant but was still very close to statistical significance (*p* = 0.0662).

The data generated for the two age groups, i.e., those aged 37.5 years or younger and those over 37.5 years, were analyzed separately to define the two age strata. A cutoff point of 37.5 years was chosen because it represents an approximate midpoint in the age range where hair loss commonly begins or becomes more noticeable [27].

As shown in Table 3, individuals who were 37.5 years old or younger did not show changes in the number and density of hairs in either treatment group (probiotic or placebo). However, individuals older than 37.5 years, when treated with probiotic, showed significant increases in both hair number and hair density (*p* = 0.0469 and *p* = 0.0495, respectively) after 16 weeks of intervention. However, there were no significant differences between the groups.

The results of the parameters related to hair length obtained by the FotoFinder technique (number and density of anagen hairs, number and density of telogen hairs, and mean hair length) did not change significantly at the end of this study in any age stratum (≤37.5 years and >37.5 years) regardless of the treatment assigned. However, it can be seen that the *p*-values approached statistical significance in the younger age group for the following parameters: number of anagen hairs, number of telogen hairs, density of telogen hairs, and mean hair length (*p* = 0.0772, *p* = 0.0693, *p* = 0.0669, and *p* = 0.0871, respectively). The latter parameter increased in the probiotic-treated group and decreased in the placebo group, whereas the other three parameters decreased in the probiotic-treated group and increased in the placebo group (Table 4).

Regarding the parameters related to hair thickness (number and density of terminal hairs, number and density of vellus hairs, and mean hair thickness) shown in Table 5, different behaviors were observed in the two age strata. Thus, in younger individuals (≤37.5), the number and density of vellus hairs increased significantly in the placebo group (*p* = 0.0314 and *p* = 0.0143, respectively) and decreased, although not significantly (*p* = 0.3746 and *p* = 0.4861, respectively), in the probiotic group. Furthermore, this behavioral difference between the two treatment groups was statistically significant (*p* = 0.0338 for both the number (*p* = 0.0338) and density of hairs (*p* = 0.0275). When comparing the change in mean hair thickness between the two treatment groups, significant differences were observed (*p* = 0.0492), with an increase in mean thickness in the probiotic group (*p* = 0.7278) and a significant reduction in the placebo group (*p* = 0.0149). Regarding the results obtained in individuals aged >37.5, only the density of terminal hairs in the probiotic-treated group showed a significant increase (*p* = 0.0593) after 16 weeks of intervention, although no significant differences were observed compared to the placebo group.

The results obtained in the comparative scalp photographs showed no differences between the treatment groups, nor did the results of the sebum measurements or manual trichograms.

### 3.2. Taxonomic Analysis

#### 3.2.1. Alpha Diversity

**Figure 2:**
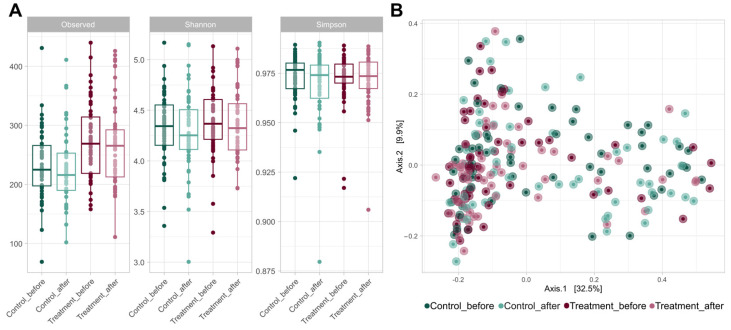
(**A**) Boxplots of the alpha diversity metrics, including the observed richness, Shannon index, and Simpson index. These metrics were calculated using ASVs (99.9% similarity threshold) for each experimental group (control and treatment) at V1 and V2. (**B**) A principal coordinate analysis (PCoA) plot based on Bray–Curtis distances at the ASV level of the gut microbiome of volunteers in both groups at V1 and V2. The axes of the plot represent the two dimensions that explained the highest proportion of variance in the communities for each analysis.

The use of probiotics did not have a significant effect on alpha diversity (Figure 2A). Although Shannon’s and Simpson’s coefficients showed no differences between the groups, the control group had significantly more ASVs than the treated group, both before (*p* = 0.001 in Wilcoxon paired test) and after treatment (*p* = 0.002). However, no significant increase was observed in any of the alpha diversity parameters evaluated after placebo or probiotic intake.

#### 3.2.2. Beta Diversity

The addition of either probiotic or placebo treatment did not affect the bacterial communities present in the samples (Figure 2B). None of the treatments caused significant changes in the overall microbiome of volunteers. However, significant differences were observed in the beta diversity level between the control and probiotic-treated volunteers (*p* = 0.001 in the PERMANOVA test).

#### 3.2.3. Microbial Communities

**Figure 3:**
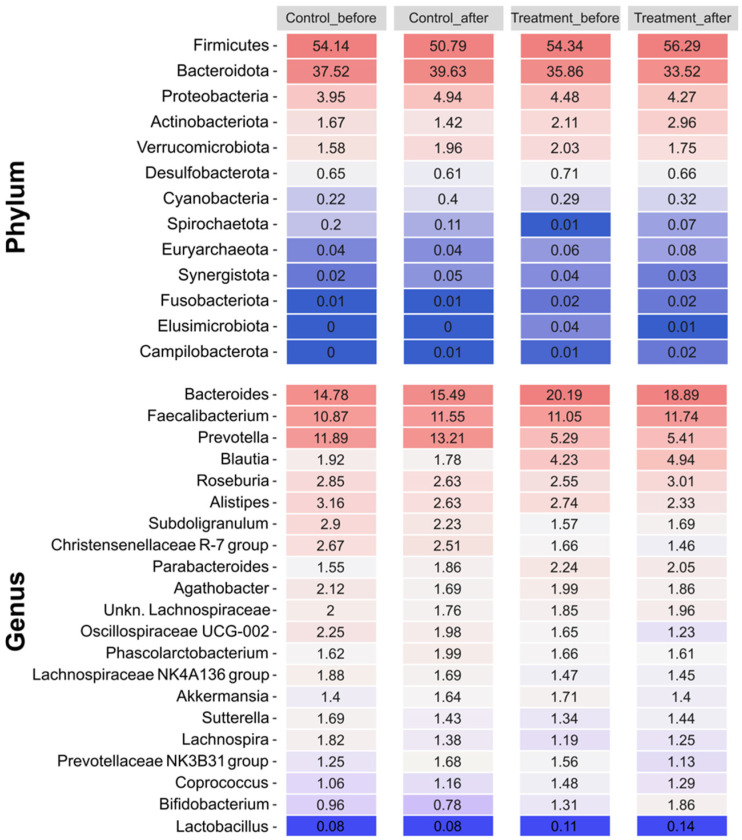
Relative abundances of the most prevalent phyla and genera (including *Lactobacillus*) present in each group (control and treatment) at each sampling point (V1, before; V2, after). The color scale represents relative abundance levels, with dark blue and dark red indicating the lowest and highest abundances, respectively.

The gut microbiome composition of each group of volunteers was consistent with the standard composition, with *Firmicutes* being the dominant phylum in all samples, accounting for slightly more than 50% of the total. *Bacteroidetes* was the second most abundant phylum, with a relative abundance greater than 35%. Other phyla, such as *Proteobacteria*, *Actinobacteria*, and *Verrucomicrobiota*, were present in much lower abundances, accounting for less than 5% of the total in the different groups (Figure 3). *Bacteroides* was the most abundant genus among the volunteers, with a seemingly higher presence in the treated group samples at the beginning and end of the treatment, although this difference was not statistically significant. The other most abundant genera in the samples were *Faecalibacterium*, *Prevotella*, *Blautia*, *Roseburia*, and *Alistipes*, with similar proportions in all the samples. No significant differences were found in the relative abundance of any genus or phylum between V1 and V2 in either the control or treatment groups. However, genera with differential abundance were found between the placebo and treated samples both before and after treatment, with most of these genera belonging to the *Lachnospiraceae* family.

**Figure 4:**
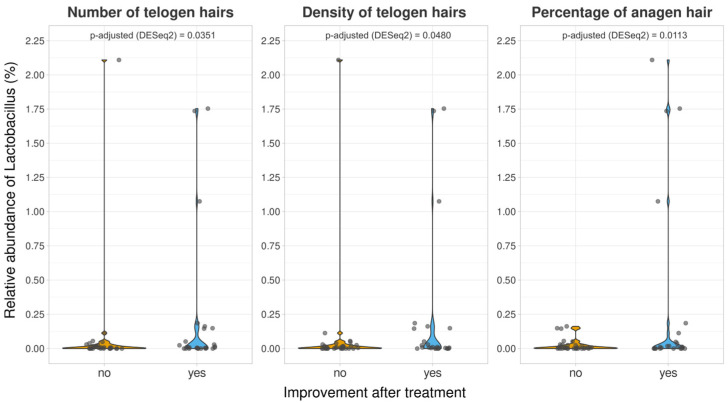
Relative abundances of the genus *Lactobacillus* for volunteers who were experimented on, with different responses to the treatments in terms of the number of telogen hairs, density of telogen hairs, and percentage of anagen hairs. *p*-adjusted derived DESeq2 differential abundance analyses were included for each comparison.

The analysis focused on *Lactobacillus*, which is the genus of the three strains in the probiotic mix. The average abundance of this genus was less than 0.2% in all groups, with a slight increase after probiotic treatment. At V2, there were no significant differences in the relative abundance of *Lactobacillus* between the two groups. Furthermore, we attempted to determine whether the improvement in each of the measured dermatological variables was accompanied by an increased presence of *Lactobacillus*. *Lactobacillus* was significantly more abundant (*p*-adjusted < 0.05 in the DESeq2 test) in the probiotic-treated volunteers, who showed improvement in three variables (number of telogen hairs, density of telogen hairs, and percentage of anagen hair) compared to those who did not experience improvement (Figure 4). However, the same analysis performed in the control group also showed a significant increase in the presence of this genus among volunteers who experienced an improvement in the percentage of anagen hair.

There was no clear correlation between the prevalence of any specific genus and the improvement in most dermatological variables, even in genera known to produce noteworthy compounds. This is an example of how certain bacteria such as Bifidobacterium can produce biotin, which is essential for preventing alopecia [28]. Furthermore, genera such as *Roseburia* and *Faecalibacterium* produce short-chain fatty acids with anti-inflammatory properties, which could also help combat hair loss [29].

### 3.3. Adverse Events (AEs)

There were no serious AEs during the study, and no AEs were found to be related to the investigational products.

## 4. Discussion

Androgenetic alopecia (AGA) is a chronic condition that causes hair loss. It affects approximately 80% of men and 50% of women [1]. The primary causes of AGA are genetic and hormonal factors [3]. In individuals with AGA, male hormones or androgens cause hair follicles to spend less time in the anagen phase and miniaturize, resulting in abnormally short and thin hair shafts [1,30].

Although AGA is a common condition, it is difficult to treat because of its chronic nature and interplay between genetic and environmental factors. Medications such as minoxidil and finasteride have been effective and can cause adverse effects that can harm patients’ quality of life. Therefore, the development of new therapies for androgenetic alopecia (AGA) treatment has led to the emergence of dietary supplements with high levels of tolerance, such as amino acids, hydrolyzed marine collagen [31], herbal extracts [14], and biotin [32]. In recent years, numerous investigations have shown that probiotics can provide beneficial health effects by modulating the intestinal microbiota and reducing dysbiosis. Thus, the usefulness of probiotics in the prevention and treatment of gastrointestinal diseases, allergies, and infections has been demonstrated, but the latest research on the intestinal microbiome, favored by the development of metagenomics, shows a clear relationship between probiotics and certain diseases, such as those related to the skin [33]. In this sense, the potential of oral probiotics as a new therapeutic approach for skin disorders such as acne, atopic dermatitis, psoriasis, rosacea [34,35,36], and hair health through the gut–skin axis stands out [37,38]. Moreover, different clinical studies conducted in individuals without serious diseases who were administered probiotics have shown that their use is safe [39]. Therefore, our clinical trial in patients with AGA aimed to provide additional evidence on the safety of probiotics by exploring their possible application in the treatment or prevention of androgenetic alopecia.

Clinical evaluation of patients suspected of androgenetic alopecia (AGA) involves determining the location and extent of hair loss, as well as the presence of inflammation (perifollicular erythema and/or scaling), using the traction technique and studying the trichograms [22]. In addition, precise techniques based on digital dermoscopy (FotoFinder) have been used to evaluate various parameters that characterize AGA and its progression.

In androgenetic alopecia, there is an alteration in the dynamics of the hair cycle comprising a gradual shortening of the length of the anagen phase and an increase in the length of the telogen phase, resulting in an increase in the number of telogen hairs and a shortening of the new anagen hairs, which determine hair length [40].

This study’s overall results showed a significant decrease in the number of telogen hairs (hair loss phase) in the probiotic group compared to that in the placebo group (reduction of 6.5%). This suggests that the patients taking probiotics experienced less hair shedding and showed a tendency to keep the number of long hairs stable compared to the placebo group. When topically applied, minoxidil stimulates the production of prostaglandin E2, which increases the duration of the anagen phase. However, it can cause side effects, such as contact dermatitis, headaches, and hypertrichosis [30]. Another drug used to treat AGA, oral finasteride, reduces the number of telogen hairs due to its antiandrogenic action [41]. Despite this, its adverse effects, including loss of libido, erectile or ejaculatory dysfunction, depression, prostate cancer, and gynecomastia, suggest the need to explore alternative treatments [3]. Other drugs, such as spironolactone, dutasteride, flutamide, and bicalutamide, are anti-androgens that block the conversion of testosterone to dihydrotestosterone. This prevents its accumulation in the hair follicle and prolongs the anagen phase but also causes adverse health effects [9,12,30].

With respect to treatment with the probiotic used in this study, although these effects are not clearly supported by statistical significance (*p* < 0.05) when comparing the two treatment groups, they can be clinically significant. This is because the significant effect on the reduction in the number of telogen hairs after 16 weeks of treatment with the probiotic tended to emulate that which has been observed in treatments with licensed drugs such as finasteride [41] (reduction of 14.6% with finasteride and 6.5% with the probiotic under study), albeit without any adverse reactions occurring.

Regarding hair thickness, this study found that the mean thickness (mm) decreased significantly in the placebo group but remained stable in the probiotic group. These results suggest that patients taking probiotics maintain the thickness of their hair, whereas those taking placebo experienced continued hair loss and miniaturization (vellus hair). These results are in line with those obtained in the only published clinical study evaluating the effects of probiotics on AGA [13]. Although the bacterial strains in that study were not *Lactiplantibacillus plantarum*, which is the probiotic used in our study, their results showed a significant improvement in hair thickness in 93% of the patients after 16 weeks of treatment.

Therefore, it can be affirmed that the probiotics used in this study tend to slow down the rate of hair miniaturization without causing adverse health effects.

When we divided the study population into those aged 37.5 years or younger and those over 37.5 years, as well as compared the effects of taking the probiotic versus the placebo, we observed different outcomes in each age group.

In the participants aged 37.5 years or younger who were treated with placebo, hair miniaturization was again observed, as was the case in the total sample of individuals in this study. The difference was significant with respect to the probiotic-treated group, which did not modify hair thickness and reduced the number of vellus hairs. These results indicate that, in the placebo group, alopecia continued to progress during the 16 weeks of the study, whereas it slowed down in the probiotic group. Some authors set the cutoff point for the age of early alopecia at 40 years [27] and others at 30 years [42]. Our sample of individuals aged 37.5 years or younger was in the age group of onset of “early alopecia,” which is influenced by genetic, hormonal, behavioral, and lifestyle factors. An important consequence for the health of young people with AGA is the development of psychological problems, such as anxiety, low self-esteem, and sexual dysfunction, significantly reducing the quality of life of individuals suffering from AGA [27,42]. Therefore, the results of our study open a new avenue for research on the safe prevention and treatment of early AGA.

For the participants over 37.5 treated with probiotics, we observed an increase in the number and density of terminal hairs after 16 weeks of treatment. This effect was not observed in the placebo group, in which there was no change in the parameters related to hair thickness. This result could indicate a tendency for hair to thicken, although no differences in thickness were observed after treatment.

It was observed that the number of telogen and vellus hairs decreased in the probiotic group but only in younger individuals and not in those older than 37.5 years. The mean hair thickness also increased in the younger age group but not in the older age group. However, the number of terminal hairs increased in the >37.5 age group, and no change was observed in the younger age group. These results suggest that the effect of probiotics may be dependent on the age at which the first signs of androgenetic alopecia appear.

The use of probiotics did not significantly change the diversity or overall composition of the microbiome in the volunteers who received probiotics. However, upon analyzing the abundance of *Lactobacillus*, it was observed that this genus was significantly more abundant (adjusted *p*-value of < 0.05 in the DESeq2 test) in volunteers who received probiotics and showed positive responses for three variables (number of telogen hairs, density of telogen hairs, and percentage of anagen hair) compared to those who did not experience improvement. However, analysis performed in the control group also showed a significant increase in the presence of this genus in volunteers who experienced an improvement in the percentage of anagen hair. This finding makes it difficult to confirm the direct effect of probiotic intake on the three dermatological variables, but it does emphasize the importance of gut lactobacilli in the treatment of alopecia.

The gut–skin axis is an emerging concept that suggests a bidirectional connection between gut microbiomes and skin health. This relationship has been investigated in the context of various skin and gastrointestinal diseases, and it is believed that gut dysbiosis may influence the onset and progression of skin diseases. Metabolites produced by the gut microbiota and their mechanism of metabolic activity may influence skin health by mediating the intestine–skin axis [43,44,45]. In this sense, a study conducted with a topical solution of inactivated *Lactiplantibacillus plantarum* in individuals with thinning hair showed that these microorganisms are producers of metabolites such as keratinocyte growth factor (KGF), which maintain the anagen phase and hair growth [17].

The presumed mechanism of action of the probiotic investigated in this study is that it is a non-biotin-consuming bacterium. Colonization of the intestine by these bacteria can compete with, and displace, biotin consumers. This results in the biotin produced, both by intestinal bacteria as a metabolite and via ingestion, are becoming more bioavailable [46]. Several studies have shown that biotin supplementation can improve hair growth [46,47].

## 5. Conclusions

In this clinical study, 150 patients were evaluated, of whom 115 completed the study. Demographic distribution showed a similar proportion of sex and age between the placebo and probiotic groups.

It can be concluded that our probiotic treatment comprising *L. plantarum* DCn_07 (CECT 30102), *L. plantarum* DCn_06 (CECT 30103), and *L. pentosus* DCn1_05 (CECT 30104) has a specific effect on improving follicular hair thickness and reducing the number of hairs in the shedding phase, avoiding their gradual miniaturization, which is a characteristic of AGA, and the microbiome analysis revealed differences in the overall microbiome between the two groups.

However, due to the complexity of the targeted pathology, the different effects observed according to age point to a need to explain the mechanism of action; thus, further clinical studies are necessary to confirm its efficacy.

## 6. Patents

Vilanova, C, Porcar, M, Morán FJ (2022), Probiotic for treating and preventing alopecia, EP 4 071 236 A1.

## Figures and Tables

**Table 1:** Hair count and density at baseline visit and after 16 weeks of treatment.

Variable		Probiotic	Placebo	*p*-Value
Hair count	Baseline	159 ± 56.1	167 ± 50.2	
	Week 16	163 ± 66.8	175 ± 61.7	0.3638
	*p*-value	0.5647	0.1423	
Hair density/cm^2^	Baseline	174 ± 58.3	184 ± 55.6	
	Week 16	178 ± 69.6	192 ± 69.3	0.5884
	*p*-value	0.9509	0.4033	
Number of telogen hairs	Baseline	34.3 ± 38.1	34.5 ± 30.4	
	Week 16	32.1± 26.9	33.1 ± 27.85	0.3638
	*p*-value	0.0314	0.3638	
Telogen hair density/cm^2^	Baseline	35.5 ± 29.7	36.7 ± 30.8	
	Week 16	35.1 ± 39.2	38.1 ± 33.7	0.6693
	*p*-value	0.2170	0.5238	
Number of anagen hairs	Baseline	83.8 ± 29.6	87.9 ± 30.5	
	Week 16	82.4 ± 30.6	90.8 ± 32	0.4177
	*p*-value	0.7842	0.3806	
Anagen hair density/cm^2^	Baseline	92.8 ± 32.8	97.3 ± 33.8	
	Week 16	91.3 ± 33.9	99.4 ± 33.8	0.5146
	*p*-value	0.7769	0.5262	

Data are presented as mean ± standard deviation. *p* < 0.05 was considered statistically significant at Visits 1 and 2, and *p* < 0.05 was the statistically significant difference between the two treatment groups.

**Table 2:** Average length and thickness at baseline and after 16 weeks of treatment.

Variable	Probiotic	Placebo	*p*-Value
Average length (mm)			
Baseline	1.64 ± 0.313	1.62 ± 0.331	0.3185
Week 16	2.81 ± 8.82	1.63 ± 0.275	
*p*-value *	0.1593	1.000	
Average thickness (mm)			
Baseline	0.0694 ± 0.0195	0.0676 ± 0.0187	
Week 16	0.0690 ± 0.0179	0.0660 ± 0.01833	0.0662
*p*-value *	0.6842	0.0301	

Data are presented as the mean ± standard deviation. * *p* < 0.05 was considered statistically significant at Visits 1 and 2, and *p* < 0.05 was the statistically significant difference between the two treatment groups.

**Table 3:** General parameters: hair count and density in the two study groups for subjects aged 37.5 years or younger at baseline and after 16 weeks of treatment.

Variable	Probiotic	Placebo	*p*-Value	Probiotic (*n* = 33)	Placebo (*n* = 34)	*p*-Value
	Participants aged 37.5 years or younger			Participants over 37.5 years of age		
Hair count						
Baseline	153 ± 58.7	171 ± 59.0	0.0772	164 ± 53.9	162 ± 40.2	
Week 16	146 ± 55.5	184 ± 75.6		180 ± 73.1	167 ± 43.3	0.3077
*p*-value *	0.3123	0.1275		0.0469	0.5664	
Hair density/cm^2^						
Baseline	165 ± 56.6	189 ± 65.3		182 ± 59.7	179 ± 44.5	
Week 16	157 ± 47.9	201 ± 85.0	0.0659	199 ± 81.0	183 ± 48.6	0.2591
*p*-value *	0.2430	0.1387		0.0495	0.6941	

Data are presented as the mean ± standard deviation. * *p* < 0.05 was considered statistically significant at Visits 1 and 2, and *p* < 0.05 was the statistically significant difference between the two treatment groups.

**Table 4:** The mean and standard deviation of the parameters related to hair length in the two study groups at baseline and after 16 weeks of treatment.

Variables	Probiotic	Placebo	*p*-Value	Probiotic	Placebo	*p*-Value
	Participants aged 37.5 years or younger			Participants over 37.5 years of age		
Number of anagen hairs						
Baseline	82.1 ± 31.8	90.7 ± 34.5	0.0772	85.5 ± 27.5	85.2 ± 26.3	
Week 16	74.8 ± 27.9	94.8 ± 37.2		89.8 ± 31.7	86.6 ± 25.5	0.4521
*p*-value *	0.1819	0.2281		0.2985	0.975	
Number of telogen hairs						
Baseline	28.1 ± 24.8	29.1± 22.4		36.2 ± 28.6	37.1 ± 32.1	
Week 16	22.9 ± 15.4	36.9 ± 39.5	0.0693	45.3 ± 49.4	31.9 ± 17.2	0.0987
*p*-value *	0.2605	0.1427		0.0521	0.6778	
Density of anagen hairs/cm^2^						
Baseline	90.9 ± 35.2	100 ± 38.2		94.7 ± 30.5	94.4 ± 29.1	
Week 16	82.8 ± 30.9	103 ± 38.5	0.1041	99.4 ± 35.1	95.9 ± 28.2	0.4509
*p*-value *	0.1525	0.3741		0.2994	0.9713	
Telogen hair density/cm^2^						
Baseline	31.1 ± 27.5	32.2 ± 24.8		40.1 ± 31.7	41.0 ± 31.7	
Week 16	25.0 ± 16.2	40.8 ± 43.7	0.0669	44.9 ± 51.1	35.4 ± 19.0	0.3174
*p*-value *	0.2456	0.1463		0.3393	0.6416	
Mean length (mm)						
Baseline	1.73 ± 0.265	1.70 ± 0.290		1.56 ± 0.340	1.55 ± 0.355	
Week 16	1.75 ± 0.220	1.63 ± 0.286	0.0871	3.84 ± 12.4	1.63 ± 0.268	0.3532
*p*-value *	0.4035	0.1097		0.1733	0.966	

Data are presented as the mean ± standard deviation. * *p* < 0.05 was considered statistically significant at Visits 1 and 2, and *p* < 0.05 was the statistically significant difference between the two treatment groups.

**Table 5:** The mean and standard deviation of parameters related to hair thickness in the two study groups at baseline and after 16 weeks of treatment.

Variables	Probiotic	Placebo	*p*-Value	Probiotic	Placebo	*p*-Value
	Participants aged 37.5 years or younger			Participants over 37.5 years of age		
Number of terminal hairs						
Baseline	86.6 ± 32.9	101 ± 41.1		90.0 ± 36.1	83.7 ± 34.2	
Week 16	76.6 ± 30.4	102 ± 46.3	0.1088	98.1 ± 39.3	87.6 ± 28.8	0.255
*p*-value *	0.1011	0.511		0.0641	0.8081	
Number of vellus hairs						
Baseline	23.6 ± 26.8	19.1± 14.2		31.7 ± 27.3	38.6 ± 41.9	
Week 16	19.2 ± 12.3	29.0 ± 30.8	0.0338	36.6 ± 41.2	30.9 ± 24.8	0.2683
*p*-value *	0.3746	0.0314		0.5384	0.3413	
Density of terminal hairs/cm^2^						
Baseline	95.9 ± 36.5	109 ± 48.7		99.6 ± 40.0	92.7 ± 37.8	
Week 16	84.9 ± 33.7	113 ± 51.2	0.0644	109 ± 43.2	97.0 ± 31.9	0.2446
*p*-value *	0.1064	0.2989		0.0593	0.808	
Density of vellus hairs/cm^2^						
Baseline	26.2 ± 29.7	21.2 ± 15.8		35.1 ± 30.2	42.7 ± 46.3	
Week 16	23.0 ± 19.6	32.4 ± 34.3	0.0275	40.5 ± 45.7	34.2 ± 27.5	0.2679
*p*-value *	0.4861	0.0143		0.5389	0.3403	
Mean thickness (mm)						
Baseline	0.0726 ± 0.0192	0.0739 ± 0.0189		0.0661 ± 0.0196	0.0614 ± 0.0165	
Week 16	0.0741 ± 0.0182	0.0670 ± 0.0154	0.0492	0.0641 ± 0.0165	0.0649 ± 0.211	0.3757
*p*-value *	0.7278	0.0149		0.6819	0.3989	

Data are presented as the mean ± standard deviation. * *p* < 0.05 was considered statistically significant at Visits 1 and 2, and *p* < 0.05 was the statistically significant difference between the two treatment groups.

## Data Availability

The original contributions presented in the study are included in the article, further inquiries can be directed to the corresponding author.

## Abbreviations

Alpha diversity refers to the variety of species within a specific area or ecosystem. In this study, it was used to measure species richness and the evenness of microbial communities within each patient sample, thus allowing for the comparison of biodiversity between the probiotic treatment and control groups.

Beta diversity was used to quantify the differences in species composition between ecosystems or along environmental gradients. It helps one to understand the species turnover, and it was also used to compare the diversity of microbial communities between the patients that received the probiotic treatment and those who received the control treatment.

The Shannon index considers both the abundance and evenness of species in a sample; as such, it provides a measure of microbial biodiversity within patient samples, accounting for how species are distributed under the probiotic and control treatments.

The Simpson index assesses the likelihood that two randomly selected individuals from a sample belong to the same species. It indicates species dominance, and it provided insight into how evenly the microbial species were represented in the communities of the patients treated with probiotics versus the control group.
